# Externalist argument against medical assistance in dying for psychiatric illness

**DOI:** 10.1136/jme-2022-108431

**Published:** 2022-09-29

**Authors:** Hane Htut Maung

**Affiliations:** Department of Politics Philosophy and Religion, Lancaster University, Lancaster, UK

**Keywords:** euthanasia, mental disorders, psychiatry, disabled persons, philosophy

## Abstract

Medical assistance in dying, which includes voluntary euthanasia and assisted suicide, is legally permissible in a number of jurisdictions, including the Netherlands, Belgium, Switzerland and Canada. Although medical assistance in dying is most commonly provided for suffering associated with terminal somatic illness, some jurisdictions have also offered it for severe and irremediable psychiatric illness. Meanwhile, recent work in the philosophy of psychiatry has led to a renewed understanding of psychiatric illness that emphasises the role of the relation between the person and the external environment in the constitution of mental disorder. In this paper, I argue that this externalist approach to mental disorder highlights an ethical challenge to the practice of medical assistance in dying for psychiatric illness. At the level of the clinical assessment, externalism draws attention to potential social and environmental interventions that might have otherwise been overlooked by the standard approach to mental disorder, which may confound the judgement that there is no further reasonable alternative that could alleviate the person’s suffering. At the level of the wider society, externalism underscores how social prejudices and structural barriers that contribute to psychiatric illness constrain the affordances available to people and result in them seeking medical assistance in dying when they otherwise might not have had under better social conditions.

## Introduction

Medical assistance in dying (MAiD) is a practice that receives legal protection in several jurisdictions, including the Netherlands, Belgium, Switzerland and Canada. The practice includes voluntary euthanasia, which is where the physician administers a fatal drug to a patient with the intention of causing death at the patient’s request and assisted suicide, which is where the physician supplies the patient with a fatal drug that is then administered by the patient with the intention of causing death. Most commonly, MAiD is provided for suffering associated with terminal somatic illness, such as advanced cancer, progressive neurological disease and major organ failure.^1^ However, some jurisdictions, including the Netherlands and Belgium, have also extended its provision for severe psychiatric illness that is deemed to be irremediable. In the Netherlands, a number of recipients of psychiatric MAiD have been increasing every year, with 13 cases in 2011, 41 cases in 2014, 67 cases in 2018 and 88 cases in 2020.^2–5^ In Belgium, a number of recipients of psychiatric MAiD have recently been decreasing, with 45 cases in 2014, 34 cases in 2018 and 22 cases in 2020, although the practice remains well established there.^6–8^

The provision of MAiD has been defended on the basis of the moral right to avoid indignity and suffering when facing a severe and irremediable illness.^9–13^ However, the practice has also been criticised on clinical and ethical grounds. Some argue that psychiatric MAiD is unjustified because it is not possible to ascertain whether or not a psychiatric illness is irremediable.^14–16^ Others criticise the practice for making it easier to terminate life in response to suffering that is in part socially mediated.^17 18^ In this paper, I consider how the argument against psychiatric MAiD is bolstered by recent work in the philosophy of psychiatry on externalism about mental disorder. Such philosophical work has led to a renewed conceptualisation of mental disorder that emphasises the role of the relation between the person and the external environment in the constitution and maintenance of psychiatric illness.^19–23^ This is significant, because it highlights that the suffering associated with a psychiatric illness may in part be socially constituted, with implications for the practice of psychiatric MAiD at the level of the clinical assessment and at the level of the wider society.

Regarding terminology, throughout the paper, I use the expression ‘psychiatric MAiD’ to refer to MAiD for psychiatric illness. However, I will only be discussing the voluntary provision of MAiD, where the patient has requested and consented to the intervention. Involuntary euthanasia, which is widely regarded as morally impermissible and a criminal offence in all jurisdictions, will not be discussed here. And so, whenever I use the expression ‘psychiatric MAiD’ in this paper, I am only referring to the voluntary provision of MAiD for psychiatric illness.

## The debate around MAiD

The bioethical argument in support of psychiatric MAiD is usually based on two considerations. The first, as noted above, is one’s moral right to avoid the indignity and suffering associated with an irremediable illness by being allowed to choose the manner and timing of one’s death. The second is the assumption of equivalence between somatic illness and psychiatric illness.^9–13^ Schüklenk writes:

Some may argue these patients should tough it out, and say ending their lives is always an inappropriate answer to their suffering. That decision, however, is one that competent patients should be able to make for themselves … Implicit in arguments made by those who might support MAID severe physical illness, but not in severe mental illness is that suffering the former is much worse than suffering the latter. Another reality check: The intensity of suffering in severe mental illness can be as terrible as that of the most severe physical conditions.^12^

The suggestion here is that people with severe and irremediable psychiatric conditions should be as eligible to seek MAiD in order to avoid indignity and suffering as people with severe and irremediable somatic conditions.

Against the above, the practice of psychiatric MAiD has been widely criticised because it is often not possible to ascertain whether or not a psychiatric illness is irremediable.^14–16^ For example, Cowley argues that the course of a somatic illness such as cancer is usually predictable enough to inform a judgement about irremediability, but the course of a psychiatric illness is too unpredictable to inform such a judgement.^14^ Furthermore, van Veen *et al* note that the perceived irremediability of the suffering may be influenced by factors such as hopelessness and treatment refusal.^16^

Some commentators also criticise MAiD for overlooking the extent to which suffering is socially mediated. Recently, this line of criticism has been centred around the passing of Bill C-7 in Canada, which extends the provision of MAiD to ‘individuals whose natural death is not reasonably foreseeable’.^24^ Wilson and Barker write:

Reports of lower quality of life are often the result of socially-mediated aspects of living with disability. It would be virtually unthinkable, we believe, for legislation to pick out any other group made vulnerable in part by their social circumstances … as the target of legislation aimed at making it easier to terminate a life under duress but whose end was not ‘reasonably foreseeable’.^17^

Similarly, Tremain writes:

It directly targets disabled people, offering government-facilitated suicide rather than providing social solutions to the poverty, isolation, disenfranchisement, and discrimination in housing, employment, and education that disabled people confront.^18^

The above line of criticism draws on the social model of disability, which underscores how people with impairments are disabled by the attitudes and structures in society that exclude them.^25^ A concern, then, is that MAiD is being offered as a solution to the harms suffered by disabled people when a more appropriate solution would be to address the social structures and practices that contribute to these harms. Indeed, in people with psychiatric conditions, there is evidence that adverse social circumstances can influence requests for MAiD.^26 27^

And so, the debate about MAiD highlights a tension between the individual right to avoid suffering and the social responsibility to avoid enacting injustice. In what is to follow, I consider how the debate regarding psychiatric MAiD is affected by the changing understanding of mental disorder in the philosophy of psychiatry. As we shall see, recent work on externalism about mental disorder highlights a challenge to the practice of psychiatric MAiD that reinforces the above critique of MAiD based on the consideration of social justice.

## Externalism and mental disorder

The traditional approach to mental disorder in psychiatry characterises it as something located within the affected individual. Usually, this is suggested to be some sort of brain state or process. As Zachar notes, many biological psychiatrists ‘claim that psychiatric disorders are best conceptualised as brain diseases’ and ‘downplay the distinction between psychiatry and neurology’.^28^ This is an internalist approach, insofar as it frames the problem as being internal to the person. Of course, the mental disorder may be the outcome of external risk factors, such as social adversity and psychological trauma, but the resulting mental disorder itself is characterised as an internal state.

Recently, some philosophers and psychiatrists have argued that the traditional internalist approach fails to capture important features of mental disorder.^19–23^ First, mental disorder is marked by intentionality. It involves reasons, beliefs, desires and other attitudes whose meanings and implications are constitutively dependent on the norms, conventions, structures and institutions of the social environment wherein the person is embedded. For example, Mirdamadi describes how the cultural significance of death in Iran, which is traceable in spiritual texts and has intensified due to the effect of war, influences the manifestations and consequences of depressive symptoms.^29^ Second, mental disorder is marked by relationality. It is shaped and sustained by dynamic and reciprocal interactions between the individual and the affordances in the social environment. For example, Fuchs describes how major depressive disorder is characterised by ‘circular causal processes’, which are feedback loops between the individual and the environment that sustain the symptoms and influence the course of the illness.^21^

In light of the above, some philosophers and psychiatrists have proposed an externalist approach to mental disorder, according to which a mental disorder is not solely located within the individual, but is constitutively dependent on the relation between the individual and the external environment.^19–23^ Different varieties of externalism differ in their details and their underlying metaphysical commitments. Nonetheless, what unites them is the proposal that an adequate understanding of mental disorder must encompass the processes between the individual and the external environment that shape and sustain it. This is not merely the claim that mental disorder is causally influenced by external factors, but the stronger claim that the external environment has a constitutive role in structuring and realising the mental disorder.

To be clear, externalism in no way suggests that the brain is irrelevant to mental disorder. Indeed, it is uncontroversial that mental disorder involves the brain. Rather, externalism is suggesting that mental disorder cannot be characterised exclusively as a brain state or process, because mental disorder is also constitutively dependent on how the brain is dynamically integrated in a wider system that is spatially distributed across the rest of the body and the environment with which the person interacts. Hence, according to externalism, a mental disorder involves the brain, but it is not a disorder of the brain. In addition to looking at the brain, a comprehensive understanding of mental disorder must acknowledge it as a process affecting a person who is embedded in a highly organised social and material setting.

Support for externalism comes from several sources. Broome and Bortolotti cite research that shows that a busy urban environment has an immediate impact on the level and content of paranoid ideation in people diagnosed with schizophrenia and people without schizophrenia.^19^ More recent research has also corroborated these findings.^30^ Fuchs cites evidence suggesting that major depressive disorder is often maintained and amplified by negative social interactions with partners, family members and work colleagues.^21^ Zachar and Kendler allude to the ways in which eating disorders are enabled and shaped by a cultural context that is marked by problematic norms and attitudes concerning the image of the feminine body.^23^ The implication is that the social environment has a significant role in the constitution and course of psychiatric illness. As we shall see, this raises an ethical challenge to the practice of psychiatric MAiD, both at the level of the clinical assessment for psychiatric MAiD and at the level of the wider society.

There is some respect in which an externalist analysis is not exclusive to psychiatric MAiD but may also apply to MAiD for somatic illness. Indeed, the suffering associated with a somatic illness can be compounded by social adversity and there is a legitimate worry that some people with somatic conditions might be offered MAiD instead of social interventions that could partly improve their circumstances.^17 18^ Nonetheless, there is also a respect in which an externalist analysis is more relevant to psychiatric illness than to somatic illness. While social adversity can certainly influence suffering associated it, the progression of a terminal somatic illness is largely the result of an unremitting internal process. This constrains the therapeutic effectiveness of any external intervention and makes the terminal prognosis more predictable.^14^ By contrast, insofar as it is marked by intentionality and relationality, the progression of a psychiatric illness is more profoundly contingent on the person’s interaction with the external environment. As Verhofstadt *et al* note, the suffering associated with psychiatric illness ‘often originates not only from medical problems, but from an interplay of various social factors and a build-up of problems throughout life’.^26^ This makes the prognosis more unpredictable in psychiatric illness and indicates a greater need for an externalist approach to understanding the suffering.

## A challenge to psychiatric MAiD

In order for a patient to be eligible for psychiatric MAiD, the clinical assessment by the attending physician must establish that the patient’s condition has no prospect of improvement and that there is no other reasonable course of action that could alleviate the suffering. This is codified in the legislation governing the practice of MAiD. In the Netherlands, the Termination of Life on Request and Assisted Suicide (Review Procedures) Act states that the attending physician must ‘be satisfied that the patient’s suffering is unbearable, with no prospect of improvement’ and ‘have come to the conclusion, together with the patient, that there is no reasonable alternative in the patient’s situation’ in order for the provision of MAiD to be legally permissible.^31^

Whether or not there is any reasonable alternative that could alleviate the patient’s suffering is contingent on various contextual factors, including the current medical and technological capabilities, the resources that are made available, and the range of possible interventions that are being considered. Insofar as externalism highlights the way in which the social environment has a role in the constitution and severity of psychiatric illness, it could draw attention to a greater range of relational and social interventions that may have been neglected under an internalist approach. Consider the following hypothetical example by Cooper, which makes a connection between externalism and the social model of disability:

Advocates of the social model say that since there would be no mobility problem in an environment with more ramps, disability should not be located within the individual wheelchair user … Now, consider a child who meets diagnostic criteria for conduct disorder but who lives in a poverty-stricken and gang-infested environment … In some cases, it might also be the case that if he were placed in a different environment, the diagnosed child would stop behaving ‘symptomatically’. In a nice foster home, where children are given food and aggression is unnecessary, he would stop fighting and shop-lifting.^20^

Given that the mental disorder is constitutively dependent on the relation between the person and the social environment, a change in the social environment may occasion a change in the symptomatology of the mental disorder.

While the aforementioned example is hypothetical, there is evidence from actual cases to indicate that the suicidal wishes associated with some mental disorders are strongly shaped by relational factors and may be mitigated by social interventions that have been somewhat neglected. Importantly, social connectedness has long been shown to be protective against suicidal thoughts and behaviours.^32–34^ This is a factor that could potentially be targeted by interventions to promote social inclusion and integration, as well as initiatives to increase access to support and improve cultural attitudes towards people who are suffering.^26 27^ Such relational factors are especially salient in vulnerable or oppressed groups. For example, transgender people comprise a marginalised community who suffer high rates of mental ill health and suicidality. However, recent research has shown that mental ill health and suicidality are not intrinsically linked to being transgender, but rather are occasioned and sustained by an environmental context marked by social prejudice, family rejection, transphobic violence and a lack of access to gender affirming healthcare. An externalist approach here could draw attention to important factors that could improve people’s conditions, such as addressing prejudice, improving social inclusion, affirming people’s identified genders, and providing accessible healthcare.^35 36^ Regarding people who seek psychiatric MAiD, sustained interpersonal difficulties have been shown to be major factors that contribute to suicidal wishes.^26 27^ In some cases, these difficulties could be targeted by psychosocial interventions that modify people’s interpersonal interactions and strengthen their social networks, such as dialectical-behaviour therapy and transference-focused psychotherapy, which have been shown to reduce suicidal wishes and behaviours in a proportion of people diagnosed with borderline personality disorder.^37^

However, there is evidence that such interventions are not being adequately provided. Research by Nicolini *et al* suggests that psychosocial interventions were not tried in 28% of people diagnosed with borderline personality disorder who received psychiatric MAiD.^38^ There are various reasons why these interventions may not have been tried. In some cases, the patients may have been deemed too unwell to engage with structured therapeutic programmes. In other cases, there may be further social barriers, such as financial, legal and housing problems, that not only contribute to the suffering, but also impede access to and engagement with therapy. As Koekkoek *et al* note in a recent review of therapy for people diagnosed with borderline personality disorder, some patients ‘have an abundance of social problems that prevent therapy taking place properly’.^39^

The above indicates that judgements that there is ‘no prospect of improvement’ and that there is ‘no reasonable alternative’ may not be considering the full range of potential interventions. For example, Blikshavn *et al* note that ‘treatment-resistant depression’ tends to be defined by a lack of response to pharmacological treatment, which risks overlooking potentially effective psychosocial interventions.^40^ Hence, an externalist approach to mental disorder presents a challenge to the practice of psychiatric MAiD by drawing attention to such psychosocial and environmental interventions that may have been neglected by an internalist approach.

Indeed, professional psychiatric associations have recognised the above as a serious problem. Notably, following a criminal trial in Belgium where three physicians were accused (and subsequently acquitted) of ‘murder by poisoning’ for allegedly providing psychiatric MAiD to a patient without establishing irremediability, recommendations were made to ensure that biological, psychological and social interventions have been attempted before judging that there is no prospect of improvement.^41^ Likewise, the guideline in the Netherlands recommends that state of the art treatment protocols must have been applied, including biological, psychological and social interventions.^42^ An externalist approach to mental disorder further accentuates this issue by emphasising the profound extent to which psychiatric illness is shaped and sustained by the person’s relation with the environment, which includes not only the immediate interpersonal and material setting but also the wider social and cultural context.

This brings us to a further challenge raised by externalism. In addition to the above challenge to psychiatric MAiD at the level of the clinical assessment, there is a wider problem concerning the normalisation of psychiatric MAiD in society. Blikshavn *et al* note:

A related worry is that when assisted dying for a subset of depressed patients has been normalized, assisted dying will have become an *available choice* accompanied by a certain way of viewing oneself and one’s plight. A wish to die does not arise in an ideological vacuum.^40^

Such a problem is again accentuated by an externalist analysis. As noted above, the behaviour associated with mental disorder is shaped and maintained by interactions between the individual and the affordances in the social environment. To normalise psychiatric MAiD in society would be to alter the affordances that are available to a person suffering from a mental illness, which could alter the person’s behaviour and the course of the illness. More specifically, providing an environmental context where MAiD is an option may result in more people seeking death to escape their suffering.

Recent research by Verhofstadt *et al* corroborates the above concern. Testimonies from people who sought psychiatric MAiD reveal that MAiD was often viewed as a ‘therapeutic’ option and an acceptable way of realising death. Sometimes, this attitude reflected a belief in the afterlife and the hope of being reunited with deceased loved ones. However, the testimonies also highlight potential external factors which, had they been available, could have prevented people from seeking MAiD. These factors include more accessible mental healthcare, more therapeutic resources for conditions that are deemed ‘difficult-to-treat’, and improved social attitudes towards people who are perceived to deviate from cultural norms.^26^ Hence, there is a concern that barriers and inequalities in the environment are constraining the possibilities available for people and making them more likely to consider MAiD.

This problem is further compounded by the understanding that the suffering for which psychiatric MAiD is being sought is itself partly shaped and sustained by the social environment. Hence, the provision of psychiatric MAiD could perpetuate social injustice by normalising the deaths of vulnerable or oppressed groups of people whose conditions are sustained by social prejudices and structural barriers. Indeed, Verhofstadt *et al* show that financial difficulties often compound the suffering associated with psychiatric illness and can even contribute to people’s decisions to request MAiD.^27^ This reveals a genuine concern that socioeconomically vulnerable people may be seeking psychiatric MAiD for suffering that is partly constituted by an unjust social context.

In addition to the concern about socioeconomically vulnerable people, research has also revealed that there is a substantial gender disparity in cases of psychiatric MAiD, with 70%–77% of cases in the Netherlands and 75% of cases in Belgium being comprised of women.^43^ Slightly different figures are suggested in a report by the Centre of Expertise in Euthanasia in the Netherlands, with 60% of requests and 64% of completed cases being composed of women.^44^ Several reasons could underlie the above gender disparity. For example, the gender disparity for psychiatric MAiD is the inverse of the gender disparity for suicide, which may indicate that fewer men request psychiatric MAiD because men are generally less likely than women to seek psychiatric input for their suicidal ideation.^43^ Further reasons are cited by Nicolini *et al*, including:

Gender-based violence, affecting 35% of women worldwide … gender-based discrimination and unfavourable social and economic circumstances, such as low employment, income inequality, low social rank and status, and the unequal division of domestic labour and care.^45^

And so, there is a concern that these factors may be neglected under an internalist approach to psychiatric illness, which may lead to more women seeking psychiatric MAiD when they otherwise might not have had under more just social conditions.

## Conclusion

The provision of psychiatric MAiD has recently been defended in the bioethical literature on the assumption that psychiatric illness can sometimes be so resistant to treatment that no reasonable therapeutic alternative is available. However, I have shown that the changing understanding of mental disorder in the philosophy of psychiatry presents a challenge to this assumption. An externalist approach to mental disorder emphasises how psychiatric illness is partly shaped and sustained by the relation between the person and the social environment. This poses ethical problems for the practice of psychiatric MAiD at two levels. First, it raises doubts about the judgements that there is ‘no prospect of improvement’ and that there is ‘no reasonable alternative’ by emphasising the need to address external contributors to suffering through psychosocial and environmental interventions. Second, by highlighting how psychiatric illness can be shaped and sustained by the social environment, it raises the concern that the provision of psychiatric MAiD may lead to people from socially oppressed groups seeking MAiD for suffering that is partly constituted by an unjust social context.

The argument I have presented here does not necessarily preclude the possibility of a situation where psychiatric MAiD is morally permissible. Nonetheless, it does indicate that the moral permissibility or impermissibility of MAiD is contingent on the range of resources that are made available to help people with persistent psychiatric illness, as well as on the wider sociopolitical context wherein the people are embedded. In a society where social prejudices and structural barriers sustain people’s suffering and constrain their possibilities, the practice of psychiatric MAiD may contribute to further social injustice by normalising death as an option for people from vulnerable groups when other alternatives may have been available under more just social conditions. This indicates the need for a serious ethical and political debate on the social responsibility to address these social harms and inequalities that contribute to the suffering associated with psychiatric illness.

## Data Availability

No data are available.
